# Alterations in the Physicochemical Properties and Antioxidant Activity during Aging of Stored Raw Garlic

**DOI:** 10.3390/foods11101390

**Published:** 2022-05-11

**Authors:** Min-Jung Kang, Jae-Ran Kang, Min Seok Woo, Dawon Kang, Jung-Hye Shin

**Affiliations:** 1 Namhae Garlic Research Institute, Namhae 52430, Korea; jung-75@hanmail.net (M.-J.K.); rani921@hanmail.net (J.-R.K.); 2 Department of Physiology and Convergence Medical Science, Institute of Health Sciences, College of Medicine, Gyeongsang National University, Jinju 52727, Korea; whitewms@naver.com

**Keywords:** alliin, black garlic, γ-glutamyl-S-allylcysteine, S-allylcysteine, storage

## Abstract

Garlic, a once-a-year crop, is mass-produced in a single event. Most of the garlic harvested during the year, unless consumed or processed immediately, should be stored. Stored raw garlic (SRG) can be used to make black garlic (BG) via aging, and storage may affect the properties and quality of the BG compared with the use of raw garlic that has not been stored. This study was performed to identify the effect of long-term storage of raw garlic on the quality of BG products. SRG was aged for 21 days at 40–86 °C for BG production. Moisture content and pH gradually decreased with the aging period. Total phenolic, total flavonoid, and fructose contents were significantly increased during the aging period. Compared with non-stored raw garlic (NSRG), alliin and S-allylcysteine (SAC) contents were 1.7-fold and 5.9-fold higher in SRG, respectively, and γ-glutamyl-S-allylcysteine (γ-GSAC) content was 2.8-fold lower in SRG. The contents of alliin and γ-GSAC reduced as the aging period of SRG and NSRG progressed. However, the SAC content of NSRG increased with aging, but the SAC content of SRG decreased or increased slightly with extended aging. The antioxidant activity was also higher in BG made from NSRG rather than SRG. These results show that the SAC content is relatively low in BG manufactured from SRG compared with NSRG. Our findings suggest that it is necessary to establish an aging method suitable for SRG in BG production with high SAC content, a representative indicator of BG.

## 1. Introduction

Garlic (*Allium sativum* L.), one of the most common spices used in many cuisines, has been produced and consumed worldwide for over 7000 years, particularly in Asia [1,2]. Garlic has also been used as a medicinal herb with antibacterial, antioxidant, anti-inflammatory, anti-cancer, anti-hyperlipidemic, and immunomodulatory activity [3,4]. However, despite its multiple health benefits, a critical drawback of garlic is its strong aroma and spicy taste [5]. These unique properties of garlic make it an unpopular choice for consumption and have led to the development of innovative processing methods such as drying, soaking, macerating, steam distillation, and heating to eliminate these properties. In particular, aged garlic and dried garlic (garlic powder) are commonly manufactured garlic products in many countries. Garlic powder, which contains alliin, has been mainly used as a food additive, and aged garlic has been used as a health supplement. Aged garlic was developed to compensate for the shortcomings of raw garlic, such as its pungent taste and aroma.

Aged garlic extract (AGE) is obtained by slicing garlic and dipping it in an aqueous ethanol solution at room temperature for 20 months without heating [6]. During this process, unstable organosulfur compounds are converted into mild and stable products via elimination of the odor-causing components. The result is an odorless garlic product containing bioactive compounds [7]. AGE contains water-soluble allyl amino acid derivatives, stable lipid-soluble allyl sulfides, flavonoids, phenols, saponins, and essential macro- and micronutrients. Of these compounds, S-allylcysteine (SAC) and S-allylmercaptocysteine (SAMC) are the main water-soluble organosulfur compounds in AGE [8,9].

Black garlic (BG) is another common processed garlic product. It is aged for 10 to 40 days under specific temperature and humidity conditions and turns black due to the Maillard reaction between the amino acids and sugars in garlic [10]. AGE is the liquid extract, whereas BG is the solid phase. BG cloves show approximately 2-fold higher SAC concentrations than BG extract [11]. BG has the advantage of a shorter manufacturing process than AGE, and as a result, valuable substances can be obtained in a shorter time. In addition, because AGE is a liquid extract, ethanol used as an extraction solvent needs to be removed, whereas BG has the advantage that it can be eaten immediately. However, although the active components (i.e., SAC and SAMC) of AGE and BG are similar, the biological activity may differ slightly because the concentration of the active components varies depending on the manufacturing process and period [12,13,14,15].

The physicochemical properties and biological activity of BG are affected by aging conditions such as temperature, humidity, and period [16,17]. Aspects of the raw garlic (RG), such as bulb size and moisture content, also affect BG production. In particular, the moisture content and active ingredients in RG directly affect the quality and biological activity of the resulting BG [18,19]. However, crops that are harvested once a year, such as garlic, are mass-produced in a single event, so they should be stored for up to a year to be eaten or used for processing. Therefore, RG stored for various periods can be used for BG production. Depending on the storage period, RG shows differences in various physicochemical components, including moisture content [20]. During RG storage, allicin content decreases while the total phenolic content (TPC) increases [21]. Changes in these components alter garlic’s functional and pharmacological properties [22], and products made from such garlic may have additional component changes.

This change in RG makes it challenging to produce BG with the same quality and contents of active components every time. So far, data have been accumulating on the changes in BG’s physicochemical properties and biological activity under various aging conditions. Different aging conditions for manufacturing BG should be applied depending on whether RG has been stored. However, it is difficult to accurately analyze the effect of RG quality on the physicochemical properties and biological activity of BG as many of the previous studies do not mention whether or not RG was stored for BG production. This study analyzed changes in the active components, particularly sulfur components, when manufacturing BG from only stored raw garlic (SRG) toward characterizing the effect of long-term storage of RG on the quality of BG products.

## 2. Materials and Methods

### 2.1. Samples and Manufacturing Protocol for BG Production

Namdo garlic cultivar (35~65 g/bulb) was grown in Namhae (Gyeongnam, Korea) from October to June. The garlic harvested between 2015 and 2016 was used in this experiment. The same cultivar and agro-technique were used. The weather conditions were similar in 2015 and 2016 (average annual temperature, humidity, and illumination of 11.7 ± 6.6 °C, 63.1 ± 7.4%, and 6.58 kW/m^2^ in 2015 and 11.7 ± 7.1 °C, 67.9 ± 11.8%, and 6.18 kW/m^2^ in 2016, respectively). SRG refers to garlic harvested in 2015 was stored at −1 °C for 8 months before use, and garlic harvested in 2016 was used in experiments with and without storage. Each 10 kg of non-stored raw garlic (NSRG) and SRG bulb was equally placed into 8 covered steel trays (300 mm × 500 mm × 150 mm). Each tray was arranged for aging in a thermo-hydrostat chamber (JSRH-500CPL, JS Research Inc., Gongju, Korea). The aging temperature for BG was gradually increased to 86 °C over the first 3 days. The temperature was then gradually lowered and maintained at 75 °C for 3 days, 70 °C until the 12th day, 65 °C until the 18th day, and 40 °C until the 21st day. During the aging period, samples (600~700 g) were collected from each tray every 3 days, and the peels were removed. Some peeled garlic was used to measure the surface Hunter’s color, and the others were all crushed and used for chemical analysis. The BG aging process experiment was performed in triplicate under the same conditions.

### 2.2. Surface Color

Garlic samples were sliced at a thickness of 10 mm after removing the peel, and the surface color was measured using a colorimeter (Ultra Scan VIS, Hunter Associates Laboratory Inc., Reston, VA, USA). The colorimeter was calibrated before measurement using a standard white reflector plate (*L* = 99.4, *a* = −0.12, *b* = 0.04). Lightness (*L*), redness (*a*), and yellowness (*b*) values of each sample were evaluated by a Hunter color system (n = 6).

### 2.3. Measurement of Moisture and pH

The moisture content was measured with 1 g of crushed garlic paste using a moisture analyzer (MB25, OHAUS, Zurich, Switzerland). Each crushed garlic was blended with 10-fold volumes of distilled water (Milli-Q, Millipore, Bedford, MA, USA). The mixture was vortexed for 1 min and then filtered using a filter paper (Advantec No. 2, Toyo Roshi Kaisha Ltd., Tokyo, Japan). The filtrates were used for the analysis of pH. The filtrate aliquots (50 mL) were measured using a pH meter (Model 720, Thermo Orion, Waltham, MA, USA).

### 2.4. Total Phenolic and Flavonoid Contents

The same filtrate for pH analysis was used for total phenolic content (TPC) and total flavonoid content (TFC) analysis. To measure TPC, phosphomolybdic acid was added to the samples, yielding a blue color upon reacting with phenolic substances, which was further measured using the Folin–Ciocalteu method [23]. Each filtrate (1 mL) was mixed with 0.5 mL of Folin–Ciocalteu’s reagent (Sigma-Aldrich Co., St. Louis, MO, USA). Three minutes later, 0.5 mL of 10% Na_2_CO_3_ solution was added to the mixture and incubated for 1 h in the dark at 22 ± 3 °C. The assay was calibrated with garlic acid (Sigma-Aldrich Co.), and a standard curve was obtained to express gallic acid equivalent (GAE) per 1 g of sample (fresh weight). TFC analysis was performed by adding 0.1 mL of 10% aluminum nitrate, 0.1 mL of 1 M potassium acetate, and 4.3 mL of ethanol to 1 mL of the sample solution, followed by incubating for 40 min in a dark room at 22 ± 3 °C [24]. Absorbance was measured at 415 nm. The TFC was calculated from the calibration curve obtained by analyzing the standard quercetin (Sigma-Aldrich Co.) and expressed as quercetin equivalent (QE) per 100 g of sample.

### 2.5. Measurement of Fructose Content

Fructose was analyzed by high-performance liquid chromatography (HPLC) using the slightly modified method of Ma et al. [25]. Distilled water (20 mL) was added to 2 g of crushed garlic and extracted with an ultrasonic extractor for 30 min. The extract was centrifuged at 1200× *g* for 10 min using a centrifuge (Combi-514R, Hanil, Seoul, Korea), filtered using a 0.45 μm syringe filter, and analyzed by (HPLC, Agilent 1260, Agilent, CA, USA). Cosmosil Sugar-D (4.6 × 250 mm, Nacalai Tesque Inc., Kyoto, Japan) was used as an analytical column, and the column temperature was maintained at 30 °C. The mobile phase with a 30:70 solution of water and acetonitrile was carried at 1 mL/min with a 10 μL injected sample. The chromatogram was detected using an evaporative light scattering detector (Agilent LT-ELSD G4128A, Agilent) and calibrated with fructose standard calibration curve.

### 2.6. Measurement of Alliin Content

The 5 g of ground garlic with 30 mL of methanol was ultrasonically extracted for 10 min. The extract was filtered through a 0.45 μm syringe filter and analyzed using HPLC (Agilent 1260) [26]. Syncronis^TM^ C_18_ analytical column (3 × 150 mm, 5 μm, Thermo Scientific, Waltham, MA, USA) was used. The mobile phase consisted of two eluents: eluent A was adjusted to pH 2.0 with 85% orthophosphoric acid in a mixture of 20 mM sodium phosphate monobasic dihydrate and 10 mM sodium 1-heptane-sulfonic acid, while eluent B was acetonitrile. A gradient elution was applied starting with 100:0 A:B (*v*/*v*) and ending with 0:100 A:B (*v*/*v*). The analytical temperature of the column was maintained at 30 °C, the mobile phase flow was 0.4 mL/min, the injection volume of the sample was 10 μL, and the wavelength of the UV detector was set to 208 nm. The alliin content was calibrated using a standard alliin (ChromaDex Inc., Irvine, CA, USA) calibration curve.

### 2.7. Measurement of S-Allylcysteine (SAC) and γ-Glutamyl-S-Allylcysteine (γ-GSAC) Content

The garlic sample (5 g) was homogenized in 45 mL of distilled water, the mixture was sonicated for 30 min and then filtered with filter paper. The filtrates were re-filtered through a 0.22 μm syringe filter, and SAC and γ-GSAC were analyzed simultaneously using HPLC-PDA-MS/MS (TSQ Quantum LC-MS/MS, Thermo Scientific, Waltham, MA, USA) [27]. Agilent Zorbax SB-C18 (4.6 × 250 mm, 5 μm, Agilent Technologies) was used as an analytical column at a flow rate of 0.7 mL/min. The solvent system consisted of two eluents: eluent A was 0.1% formic acid solution, and eluent B was 0.1% formic acid dissolved in acetonitrile. A gradient elution was employed starting with 99:1 A:B (*v*/*v*) and ending with 0:100 A:B (*v*/*v*). SAC (Sigma-Aldrich Co.) and γ-GSAC (US Pharmacopeia, Rockville, MD, USA) standards were assayed under the same conditions as the samples, and the retention times were compared to calculate the SAC and γ-GSAC contents from the respective calibration curves.

### 2.8. Preparation of Water-Soluble Garlic Extracts

Crushed garlic was suspended with ten volumes of distilled water. The suspended garlic was extracted for 5 h at 70 °C. The water extract was filtered twice through four pieces of cheesecloth (Kavon Filter Products Co., Farmingdale, NJ, USA), freeze-dried, powdered, and stored at −20 °C until further analysis. At the time of the experiment, the dried material was dissolved in distilled water at a concentration of 10 μg/mL.

### 2.9. Measurement of ABTS and DPPH Radical Scavenging Activity

The 2,2-azinobis-(3-ethylbenzo-thiazoline-6-sulfonate) (ABTS) and 1,1-diphenyl-2-picrylhydrazyl (DPPH) radical-scavenging activities of garlic extracts were evaluated according to previous protocols [28]. Briefly, ABTS radical cation (ABTS^+^) solution was prepared by reaction of 7 mM ABTS solution with 2.4 mM potassium persulfate overnight in the dark at room temperature. The concentration of the ABTS^+^ solution was adjusted to an absorbance of 1.5 at 415 nm. Equal quantities of garlic extract (0.5~2.0 mg/mL) were mixed with ABTS^+^ (100 μL) and reacted at room temperature for 5 min. For measuring DPPH radical-scavenging activity, a freshly prepared DPPH solution (5 mg/100 mL in ethanol) was added to each extract (0.5~4.0 mg/mL). After shaking, the mixture was incubated for 10 min in darkness. Absorbance was measured at 415 and 525 nm for ABTS and DPPH, respectively, using a microplate reader (VICTOR X3, Perkin-Elmer Inc., Waltham, MA, USA).

### 2.10. Measurement of Reactive oxygen Species (ROS) Generation in Cells

Intracellular ROS generation was measured using dichlorodihydrofluorescein (H_2_DCFDA, Thermo Fisher Scientific, Waltham, MA, USA). Chang liver cells (2 × 10^4^ cells/mL) were plated in a 96-well black plate (Thermo Fisher Scientific, Roskilde, Denmark) and a poly-L-lysine-coated confocal image dish (SPL, Pocheon, Gyeonggi, Korea). The cells were cultured for 24 h, and the garlic extracts and SAC were pretreated for 1 h prior to treatment with H_2_O_2_. The cells were incubated with 5 μM H_2_DCFDA at 37 °C for 30 min in the dark. The medium was removed, and the cells were washed three times with PBS. The fluorescence of cells in 96 wells was measured with a GloMax^®^ Explorer Multimode Microplate Reader (Promega, Madison, WI, USA) at 485 nm excitation and 535 nm emission. The fluorescence of the cells in the confocal image dish was observed using a confocal laser scanning microscope (Olympus, Tokyo, Japan) with a filter set for fluorescein isothiocyanate (FITC).

### 2.11. Statistical Analysis

Each assay was replicated at least three times. The data are presented as the mean ± standard deviation (SD). The data were analyzed by one-way ANOVA followed by Duncan’s multiple range test using SPSS (v 18.0; IBM Co., Endicott, NY, USA) after checking normality and homogeneity of variance test. Differences were considered to be statistically significant at *p* < 0.05.

## 3. Results

### 3.1. Changes in Hunter’s Color Value during Garlic Aging

**Figure 1:**
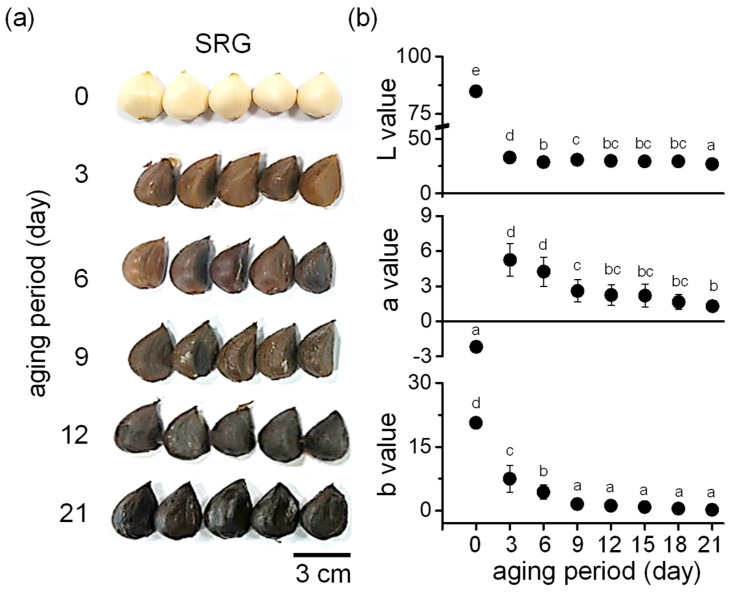
Surface color change during aging of SRG. (**a**) Representative photographs showing the color change in garlic during aging. SRG was aged for 21 days at 40–86 °C. (**b**) Analysis of lightness (*L*)*,* redness (*a*), and yellowness (*b*) values of SRG during the aging period. Data are represented as mean ± SD. Different letters indicate significant differences between the same columns based on Duncan’s multiple range test (*p* < 0.05).

In the process of BG production, the color of garlic changed rapidly on the third day of aging and gradually turned dark brown, and most of the garlic had turned black by the ninth day of aging. On the 12th day of aging, the entire garlic had turned black, and this was maintained until the 21st day when aging was completed (Figure 1a). The color was analyzed using a colorimeter. As shown in Figure 1b, the lightness (*L*) value dramatically decreased on the third day of aging. The redness (*a*) value continuously decreased during the aging period, ranging from 0.9 to 1.8 on the 21st day. The tendency toward yellowness (*b*) showed a similar trend to redness in gradually decreasing up to the last (21st) day.

### 3.2. Changes in Moisture and pH

Moisture content decreased by approximately 34–37% within the first 3 days of aging (47.4~47.7 g/100 g) and gradually reduced to about 44% by the 21st day (Table 1). The final moisture content was 44.40 ± 0.74 g/100 g in BG made from SRG. As shown in Table 1, the pH gradually decreased with the aging period. Between 3 and 9 days of aging, the pH decreased sharply, reaching close to 5.0, and then gradually decreased to 4.51 ± 0.12 in BG made from SRG during the remainder of the aging period.

### 3.3. Changes in Chemical Parameters during Garlic Aging

**Figure 2:**
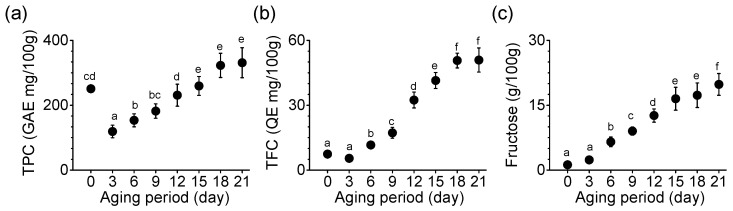
Changes in (**a**) TPC, (**b**) TFC, and (**c**) fructose content during the aging of SRG. Each point represents the mean ± SD (n = 6). Based on Duncan’s multiple range test, different letters indicate significant differences between the same sample (*p* < 0.05).

TPC, TFC, and fructose content were compared in the process of producing BG. TPC increased significantly until the 15th day of aging (*p* < 0.05), which was maintained until the end of the aging (*p* < 0.05, Figure 2a). TPC measured on the 21st day of aging was 331 GAE mg/100 g in SRG. The rate of increase in TFC was 9.3-fold in SRG on day 21 compared with day 3 (Figure 2b). TFC did not differ significantly between days 18 and 21. Fructose content increased continuously during the aging period (Figure 2c). The fructose content on the 21st day was 19.82 g per 100 g, which was approximately 8.4-times higher than on the third day. On the final (21st) day, BG made from SRG showed a significant difference from the first day of aging in TPC, TFCs, and fructose content (*p* < 0.05, Figure 2).

### 3.4. Changes in Sulfur Compounds during Garlic Aging

**Figure 3:**
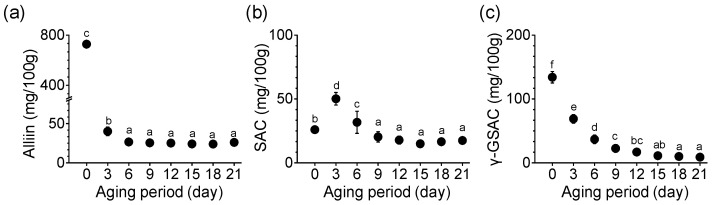
Changes in (**a**) alliin, (**b**) SAC, and (**c**) γ-GSAC content during the aging of SRG. Each point represents the mean ± SD (n = 6). Different letters indicate significant differences between the same sample based on Duncan’s multiple range test (*p* < 0.05).

The amounts of alliin, SAC, and γ-GSAC, the main sulfur compounds of garlic, changed during the aging period (Figure 3). Alliin content in SRG before aging (day 0) was 727.56 mg/100 g (Figure 3a). Alliin content decreased rapidly with the onset of the aging process, and the alliin content on day 3 of aging was 40.06 mg/100 g. This significant decrease persisted until day 6 of aging and was 26.60 mg/100 g. As shown in Figure 3b, the SAC content in SRG started at 25.93 mg/100 g and increased rapidly on day 3 to 50.14 mg/100 g. This content was 1.9-fold higher on the third day than that on the day 0, but on the sixth day it was significantly reduced to 63.3% compared with the value on the third day. On the final 21st day, the SAC content (17.46 mg/100 g) was significantly lower than that on day 0 (*p* < 0.05). The level of γ-GSAC, a precursor of SAC, showed a pattern of change similar to that of alliin (Figure 3c). The content of γ-GSAC was highest in SRG (133.99 mg/100 g) before aging. γ-GSAC was significantly decreased by the 15th day of aging (*p* < 0.05). In particular, on the third day of aging, γ-GSAC sharply reduced to 68.84 mg/100 g in SRG. Until the sixth day of aging, the reduction rate in γ-GSAC content was 51~64% compared with that before aging. The γ-GSAC content was the lowest on day 21. The content of γ-GSAC decreased as the aging period elapsed (*p* < 0.05).

### 3.5. SAC Content Shows a Lower Increase in BG Made from SRG than from NSRG

To determine if the SAC content actually decrease when BG is made from SRG rather than NSRG, BG was manufactured under the same conditions using SRG harvested in another year (2016) and then compared with the component values of BG made from non-stored RG (NSRG) harvested that year. As shown in Table 2, alliin content in garlic before aging (day 0) was 804.40 and 471.91 mg/100 g in SRG and NSRG, respectively. The alliin content decreased significantly with the onset of the aging process, and the alliin content in SRG and NSRG on day 21 of aging was 19.02 and 21.99 mg/100 g, respectively. There was no significant difference between SRG and NSRG on day 21 (*p* > 0.05). The SAC content in SRG and NSRG started at 22.35 and 3.79 mg/100 g and increased on day 21 by 1.44- and 12.45-fold, respectively. On day 21, the final content of SAC in NSRG was 1.47-fold higher than that in SRG (47.20 vs. 32.12 mg/100 g). The content of γ-GSAC was high in SRG (130.23 mg/100 g) and NSRG (368.40 mg/100 g) before aging but decreased on day 21. The contents of alliin, SAC, and γ-GSAC in NSRG were higher than that in SRG (*p* < 0.05).

### 3.6. Antioxidant Activity of BG Made from SRG and NSRG

**Figure 4:**
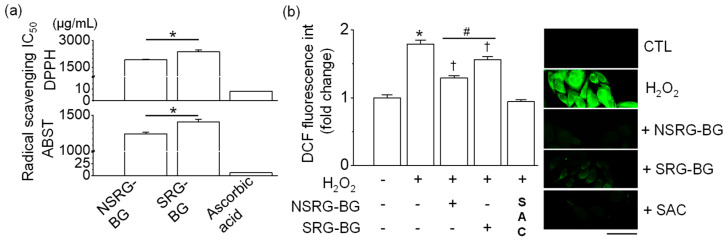
Antioxidant activity of BG made from SRG and NSRG. (**a**) DPPH and ABTS radical scavenging IC_50_. (**b**) H_2_O_2_-induced ROS generation. Chang cells were exposed to 500 μM H_2_O_2_ for 24 h after pretreatment with BG for 1 h. Green fluorescence represents ROS generation. NSRG-BG and SRG-BG represent BG made from NSRG and SRG, respectively. A BG extract mixture made from SRG harvested in 2015 and 2016 was used as SRG-BG. Ascorbic acid (2.5–10 μg/mL) and SAC (1 mM) were used for a positive control for each experiment. Each bar represents mean ± SD of four independent experiments. * *p* < 0.05 compared to each corresponding control. ^†^ *p* < 0.05 compared to H_2_O_2_ treatment. ^#^ *p* < 0.05, NSRG-BG vs. SRG-BG. NSRG-BG and SRG-BG indicate BG made from NSRG and SRG, respectively.

BG extracts decreased the content of DPPH and ABTS chemical radicals. The half-maximal inhibitory concentration (IC_50_) of the antioxidant capacity of BG made from SRG and NSRG was 2365.32 ± 119.35 and 2128.06 ± 34.41 μg/mL for DPPH and 1404.43 ± 40.94 and 1240.84 ± 24.04 μg/mL for ABTS, respectively (Figure 4a, n = 3). Chang liver cells were pretreated with BG extracts for 1 h prior to 500 μM H_2_O_2_ treatment for 24 h. High amounts of ROS were detected in cells treated with H_2_O_2_ for 24 h compared with the control. The level of H_2_O_2_-induced ROS generation was significantly decreased in cells pretreated with BG extracts by approximately 30% (n = 4, *p* < 0.05, Figure 4b). The reduction in ROS generation was higher in BG extracts made from NSRG than those from SRG.

## 4. Discussion

This study reports that the final BG manufactured from SRG had reduced or slightly increased SAC content compared with pre-aged SRG (day 0). Most of the previous studies related to BG focus on changes in the components during the BG manufacturing process and the development of stable aging methods, regardless of whether or not RG, the raw material of BG, is stored. Physicochemical changes in garlic can induce alterations in garlic function. In BG, SAC is used as a representative indicator material. SAC exhibits antioxidant properties in chronic stress-induced toxicity in vivo [29] and hepatoprotective effects in acute and chronic liver injury [30]. However, the amount of SAC was significantly lower in BG made from SRG than in that made from NSRG. According to our study, the positive health effects of BG exerted by SAC will not be as high when it is made from SRG. Therefore, it is necessary to establish an aging method suitable for SRG to produce BG with a high SAC content.

### 4.1. Alterations in Physiochemical Properties during BG Manufacturing Process

Earlier studies have demonstrated that garlic’s physicochemical properties and active components change during the BG manufacturing process [10,13,14,16]. The Maillard reaction occurs when the heating process for garlic aging begins. There is an increase in the content of organic acids, such as acetic acid, fumaric acid, malic acid, and citric acid, produced from decomposed carbohydrates [31,32]. Increased levels of browning compounds and organic acids cause a reduction in pH, prompting the breakdown of proteins, peptides, and polysaccharides. These reactions are interconnected and produce the black color, jelly-like texture, and sweet and sour taste of BG [33,34]. In addition to many physical parameters, moisture content can also affect the jelly-like texture of BG. BG has a soft and elastic texture when the moisture content reaches 40–50 g/100 g [31]. The final moisture content of BG produced in this study was approximately 44 g/100 g for SRG, indicating that the BG has a soft and elastic texture. The softening of garlic promotes the release of phenolic compounds during the heat treatment for aging. The TPC is higher in BG than in RG [35]. The TPC is increased by steam application but decreased under higher pressure or prolonged heating [36]. The increase in TPC is due to a rise in the free form of phenolic and flavonoid compounds during heating [37]. The TPC in BG can be affected by several factors, such as heating conditions (temperature, pressure, time, humidity, etc.) and duration of aging.

Polysaccharides are decomposed into reducing sugars during the BG manufacturing process and consumed through participation in the Maillard reaction [31]. In the early stage of aging, the consumption of reducing sugars is lower than the decomposition, so the sugar content is increased. However, in the late stage of BG aging, the sugar content decreases because the increasing rate of sugar from the degradation of fructans is slow. Another reason is because of the high consumption rate involved in the Maillard reaction. Polysaccharide degradation of the cell wall occurs under acidic conditions or high temperatures, resulting in a soft texture and adding jelly-like properties to BG [32]. Acidification also promotes the further hydrolysis of fructan to glucose and fructose. The complete hydrolysis of fructan during BG aging increases fructose and glucose contents, making BG sweeter, and is involved in the Maillard reaction [36]. Fructose, the main saccharide in BG, contributes to the sweet taste of BG [37].

Heat treatment is the most influential factor in increasing TPC, TFC, and fructose content in the BG manufacturing process. The first reaction upon the thermal treatment of garlic is the decomposition of alliin. The alliin content shows a linear relationship with the logarithm of the alliin concentration and heating conditions. Even under the condition of alliinase inactivation, the reaction rate is higher at high temperatures and a long processing time [33]. In garlic extract processed at 100 °C for 20 min, alliin is decreased to 84.0% [38]. These results indicate that the content of alliin is reduced by heat treatment during the aging process. Allicin, the primary substance of RG, is dramatically lower in BG than in RG as it decreases rapidly at the beginning of heat treatment [31]. Allicin is no longer an essential functional substance in BG. In the present study, BG showed a black color, sweet and sour taste, and soft and jelly-like texture due to the Maillard reaction, 44% moisture, acidification, increases in fructose content, TPC, and TFC, and a decrease in alliin content caused by heat treatment. These findings are similar to those of previous studies.

SAC generally increases by 5~6-fold during the aging period and is formed through two pathways. The simplest pathway is the enzymatic hydrolysis of γ-GSAC, which is catalyzed by endogenous γ-GTP, where one molecule of GSAC yields one molecule of SAC [39,40,41]. The SAC content in BG is affected by heating temperature, water loss, and the activation of γ-GTP. The high heating temperature contributes to the increase in the SAC content in the early stages of the BG manufacturing process, the mechanism of which is influenced by unknown factors [17]. The long-term maintenance of a high temperature is required for BG production, which inactivates γ-GTP. In addition, the process is not characterized by an appropriate pH range in which γ-GTP may be activated because BG gradually becomes acidic with aging. On the other hand, SAC is formed, indicating that SAC is converted even when γ-GTP is not involved. SAC can be produced from alliin by direct thermal treatment and from the decomposition of γ-glutamyl-S-alk(en)yl cysteine, even when γ-GTP is inactivated [15,21]. γ-GTP is completely inactivated by heating at 78 °C for 15 s, and reductions in γ-GTP activity slow SAC formation [42]. As a result of decreased alliin and GSAC content and increased γ-glutamyl-S-alk(en)yl cysteine content, the SAC content of the BG may increase during the aging process. The increase in sulfur compounds in the late aging period after γ-GTP inactivation can also be explained by this pathway. Consistent with the results of previous studies, our study shows that alliin and γ-GSAC contents decrease with progression of the BG manufacturing process, resulting in a dramatic increase in the SAC content of the final BG made from NSRG. However, the SAC content was not dramatically increased in the final BG made from SRG. It is thought that storage already increases the SAC content, and as such, it barely changes during the BG manufacturing process applied in this study.

### 4.2. Alterations in Physiochemical Properties during RG Storage

Previous studies have reported that the content of organosulfur compounds in garlic changes during cultivation and storage. The quality and biological activities of garlic are markedly affected by different storage conditions [43]. The storage period of garlic affects the chemical structure of garlic bulbs and the antioxidant capacity of garlic by increasing the content of phenolic and organosulfur compounds such as SAC and SAMC [20]. The variety and storage process significantly affect TPC of RG [22]. Garlic stored for 150 days at different temperatures (−3 °C, 4 °C, and 23 °C) shows a decrease in γ-GSAC content and an increase in alliin content at all storage temperatures [44]. Consistent with previous studies, phenolic, flavonoid, alliin, and SAC contents tended to be high in SRG. In this study, SRG refers to garlic stored at −1 °C for 8 months. The effect of storage period and storage temperature on RG components was not investigated here, and further study is needed to characterize these effects. Comparing the sulfur compounds between SRG and NSRG, the alliin and SAC contents were high in SRG, and γ-GSAC content was high in NSRG. The decrease in γ-GSAC in SRG is likely to result from the conversion of γ-GSAC, γ-glutamyl peptide, and γ-glutamyl-S-(trans-1-propenyl)-L-cysteine (γ-GSPC) to alliin, isoalliin, and cycloalliin due to the activation of γ-glutamyl transpeptidase (γ-GTP) during storage. γ-GSAC, present in large amounts in RG, is rapidly converted to alliin during the storage period by dormancy destruction, hydrolysis, dehydration, and oxidation [44,45,46]. In addition, γ-GSAC is converted to SAC via a catabolic pathway other than the alliin-allicin pathway [47].

### 4.3. SAC Content Affected in BG Made from SRG

In previous studies, the amounts of active components of BG reported by researchers have shown great variation, even for BG produced under the same conditions. The reason is thought to be that the properties of RG, the starting material for preparing BG, have not been considered in the studies. In commercialization, it is crucial to maintain a constant concentration of active components, thus meeting the quality standards for BG. Garlic is a crop that is harvested in large quantities once a year and is then stored and eaten throughout the year. Therefore, it is necessary to analyze and compare the properties of stored and non-stored garlic. However, if SRG and NSRG are to be considered for garlic harvested in the same year, the same BG manufacturing process cannot be applied at the same time. In addition, if the experiment is to be conducted simultaneously, garlic harvested in different years must be used as SRG and NSRG. Therefore, in this study, we decided to first analyze the physicochemical properties and active ingredients of BG made only from SRG. A recent study demonstrated that the RG variety and storage time influence the physicochemical and antioxidant properties of the derived BG [34]. However, the authors did not analyze the active components in BG, such as alliin, SAC, and γ-GSAC.

The contents of alliin and SAC were higher in SRG compared with NSRG, but SAC was lower in the final BG made from SRG compared with that from NSRG, and alliin maintained a very low level with no significant difference between BG made from SRG and NSRG. SAC is converted from alliin and GSAC during storage or aging. The conversion of GSAC, which shows a high content in NSRG, to SAC occurs rapidly with the onset of the BG manufacturing process, and alliin is converted to SAC as aging progresses. It is thought that the SAC content in BG produced from NSRG may be higher than in BG produced from SRG as a result of these metabolic processes.

SAC content was used for the standardization of BG because it increased the most among many ingredients in BG and was well maintained during the aging period. However, in this study, the final BG made from SRG had reduced or slightly increased SAC content compared to pre-aged SRG. The content of SAC rose on the third day of the BG manufacturing process. To standardize the SAC content of commercial BG products, it is essential to establish conditions with high and constant SAC concentration. For the production of BG, the storage of garlic should first be considered. There is a need to establish a new aging method in which a high amount of SAC is maintained in the production of BG from SRG.

## 5. Conclusions

The moisture content, pH value, and alliin and γ-GSAC contents decreased, whereas TPC, TFC, and fructose content increased with prolongation of the aging period of SRG. The SAC content of the final BG made from SRG decreased or slightly increased compared with the pre-aged SRG. However, SAC content and antioxidant activity were higher in BG made from NSRG than in BG made from SRG. Our findings suggest that it is necessary to establish an aging method suitable for SRG for the BG production with a high SAC content, a representative indicator of BG, because SAC varies depending on whether or not garlic is stored during the BG manufacturing process.

## Figures and Tables

**Table 1:** Changes in moisture content and pH in BG made from SRG during the aging period.

Aging Periods (Days)	Moisture (g/100 g)	pH
0	72.55 ± 0.19 ^d^	6.66 ± 0.03 ^f^
3	47.40 ± 1.76 ^c^	5.81 ± 0.15 ^e^
6	47.50 ± 1.61 ^c^	5.40 ± 0.11 ^d^
9	46.65 ± 1.14 ^c^	4.97 ± 0.11 ^c^
12	46.15 ± 1.14 ^bc^	4.85 ± 0.18 ^bc^
15	45.10 ± 0.70 ^ab^	4.77 ± 0.15 ^b^
18	44.75 ± 0.87 ^a^	4.70 ± 0.09 ^b^
21	44.40 ± 0.74 ^a^	4.51 ± 0.12 ^a^

Data are represented as mean ± SD. Superscript letters indicate significant differences between the same columns based on Duncan’s multiple range test (*p* < 0.05).

**Table 2:** Effect of postharvest storage and BG aging on content of alliin, SAC, and γ-GSAC.

Sulfur Components		Alliin (mg/100 g)		SAC (mg/100 g)		γ-GSAC (mg/100 g)	
Aging Period (Day)		0	21	0	21	0	21
Garlic Condition (harvest year)	NSRG (2016)	471.91 ± 9.10 ^a,^*	21.99 ± 1.27 ^b,ns^	3.79 ± 0.11 ^a,^*	47.20 ± 6.46 ^b,^*	368.40 ± 7.96 ^a,^*	56.41 ± 7.36 ^b,^*
	SRG (2016)	804.40 ± 10.51 ^a^	19.02 ± 0.34 ^b^	22.35 ± 0.33 ^a^	32.12 ± 2.91 ^b^	130.23 ± 5.86 ^a^	24.83 ± 2.26 ^b^

SRG and NSRG harvested in 2016 were aged for 21 days at 40~86 °C in the same manner. SAC, S-allyl cysteine; γ-GSAC, γ-glutamyl-S-allyl-L-cysteine; NSRG, non-stored raw garlic; SRG, stored raw garlic. The data are presented as the mean ± standard deviation (SD). Lowercase letters indicate significant differences between the same sample based on Duncan’s multiple range test (*p* < 0.05). The asterisk (*) symbol indicates a significant difference between SRG and NSRG of the same aging date. The ns indicates no significant difference between SRG and NSRG of the same aging date.

## Data Availability

No new data were created or analyzed in this study. Data sharing is not applicable to this article.
